# Arterial Hypertension Is Characterized by Imbalance of Pro-Angiogenic versus Anti-Angiogenic Factors

**DOI:** 10.1371/journal.pone.0126190

**Published:** 2015-05-07

**Authors:** Natalia Marek-Trzonkowska, Anna Kwieczyńska, Magdalena Reiwer-Gostomska, Tomasz Koliński, Andrzej Molisz, Janusz Siebert

**Affiliations:** 1 Laboratory of Immunoregulation and Cellular Therapies, Department of Family Medicine, Medical University of Gdańsk, Gdańsk, Poland; 2 Department of Clinical Immunology and Transplantology, Medical University of Gdańsk, Gdańsk, Poland; 3 Department of Family Medicine, Medical University of Gdańsk, Gdańsk, Poland; University of Bari Medical School, ITALY

## Abstract

**Objective:**

Hypertension is the most common cardiovascular disease and the main risk factor for stroke, peripheral arterial disease, arterial aneurysms and kidney disease. It has been reported recently that hypertensive patients and animals are characterized by decreased density of arterioles and capillaries in the tissues, called rarefaction. Rarefaction significantly increases peripheral resistance which results in elevated blood pressure, leads to vessel damage and induction of inflammation. Therefore, we hypothesized that hypertension is associated with decreased serum concentration of physiological pro-angiogenic factors and concomitant increased production of angiogenesis inhibitors.

**Materials and Methods:**

82 patients diagnosed with hypertension and 34 healthy volunteers were recruited to the study. Flow cytometry and enzyme-linked immunosorbent assay (ELISA) techniques were used to measure serum levels of the following cytokines: endostatin, vascular endothelial growth factor (VEGF), interleukin 8 (IL-8), angiogenin, and basic fibroblast growth factor (bFGF).

**Results:**

Hypertensive patients were characterized by increased serum concentration of endostatin which is an anti-angiogenic factor. In addition, hypertension was associated with decreased levels of physiological pro-angiogenic mediators such as: angiogenin and bFGF. The hypertensive group was also characterized by elevated levels of CRP, VEGF and IL-8 that are the hallmarks of inflammation.

**Conclusions:**

Presented results show that hypertension is characterized by imbalance of pro-angiogenic and anti-angiogenic factors in the background of inflammation.

## Introduction

Arterial hypertension is the most common cardiovascular disease. It is well known that it is associated with endothelial dysfunction [1] and structural alterations in blood vessels. Thickening and remodeling of artery walls, as well as increase in the wall-to-lumen ratio are features of the hypertension [2–4]. Nevertheless, an important role in the pathophysiology of hypertension is played by changes in microcirculation. Loss of pre-capillary arterioles and capillaries, known as microvascular/capillary rarefaction is another hallmark of the disease. In addition, it has been found that angiogenesis alterations during fetal development lead to underdevelopment of microvasculature and thus predispose to hypertension in later life [4]. Significance of effective angiogenesis for normotension maintenance has been also confirmed by oncological studies. Hypertension was found to be the common side effect of anti-angiogenic therapies aiming to stop sprout of tumor vessels [5].

Nevertheless, while some groups show that hypertension is associated with impaired angiogenesis [6], other studies report that hypertensive patients are characterized by increased serum levels of certain angiogenic factors [7]. Therefore, the aim of our study was to verify this issue. Development of new vessels is a complex process regulated by multiple interplaying factors [8], therefore we analyzed a group of mediators known to influence this phenomenon. As hypertension is strictly associated with inflammation [9–11], besides potent physiological regulators of angiogenesis such as angiogenin, endostatin or basic fibroblast growth factor (bFGF) [12–21], we studied mediators which activity is significantly increased during inflammation like vascular endothelial growth factor (VEGF) and interleukin 8 (IL-8) [22–32].

## Methods

### Patients

A total of 82 consecutive patients with arterial hypertension treated at the Department of Family Medicine of Medical University in Gdansk in Poland were recruited. The control group comprised 34 healthy normotensive volunteers matched for age and sex. Hypertension was defined according to the European Society of Hypertension and European Society of Cardiology as blood pressure ≥ 140/90 mmHg [33]. The normotension criterion was blood pressure ≤ 139/89 mmHg. Hypertension was diagnosed on the basis of results of 24h blood pressure measurement. All of the hypertensive patients were treated with hypotensive drugs for median period of 6.5 years (Table 1). The values of blood pressure used for the calculations in the study are means of 2 consecutive measurements after 20 minute rest in sitting position performed at the day of blood drawing.

**Table 1:** Clinical and laboratory characteristics of the investigated groups.

Parameter	Hypertension	Healthy
n (female/male)	82 (54/28)	34 (22/12)
Age [years]	71.5 (19–94)	62.5 (25–91)
Duration of the disease [years]	6.5 (0.5–50)	N/A
SBP [mmHg]	139.5 (108–165)	120 (100–139)
DBP [mmHg]	80 (60–105)	76 (55–80)*
Number of patients with well controlled hypertension	60 (73%)	N/A
CRP [mg/L]	1.6 (0.1–13.94)	0.9 (0.20–2.7)*
Creatinine [mg/dl]	0.83 (0.59–1.65)	0.78 (0.59–0.95)
GFR [ml/min/1,73m^2^]	84.46 (39,57–149,17)	96.15 (91–116.81)*
HbA1C [%]	5.75 (4.73–6.62)	5.60 (4.77–5.87)
Total serum protein [g/l]	72 (65–88)	73 (52–94)
TCh [mg/dl]	214 (134–355)	218 (176–273)
TG [mg/dl]	107.5 (45–364)	95 (51–252)
HDL [mg/dl]	55 (26–93)	58 (41–98)
LDL [mg/dl]	135.5 (27–263)	130 (105–195)
†Microalbuminuria (% of patients per group)	58%	0%
Weight [kg]	70 (40–125)	65 (40–100)
Hight [cm]	164 (142–195)	165 (152–186)
Waist circumference [cm]	90 (60–130)	85 (60–102)
BMI	25.65 (17.52–37.87)	23.7 (18.82–33.46)*
#Hypotensive drugs (% of treated individuals per group)	ACEI 40.9%	N/A
	β-blocker 28.6%	
	Diuretic 26.1%	
	Ca-blocker 9.6%	
	Sartan 13.0%	
Statins (% of treated individuals per group)	12.1%	14.7%

As indicated by the distribution of the variables data are presented as medians with minimum and maximum values within parentheses; n = number of subjects, SBP = systolic blood pressure, DBP = diastolic blood pressure, GFR = glomerular filtration rate, HbA1c = glycated hemoglobin, TCh = total cholesterol, TG = triglycerides, LDL = low-density lipoprotein, HDL = high-density lipoprotein, N/A = non-applicable, ACEI = angiotensin-converting-enzyme inhibitor;

^#^ hypertensive patients were on mono- or polytherapy, proportions of the patients treated with a particular group of drugs are presented as %;

^†^ microalbuminuria was defined as >3 mg/dl in spot urine albumin specific-dipstick; statistically significant differences (p< 0.05) between the groups were marked with “*”.

Patients suffering from infectious, neoplastic and autoimmune diseases, as well as individuals with secondary hypertension were excluded from the study. Other exclusion criteria were: active or passive smoking, heart failure and other cardiac complications of hypertension, hormonal replacement therapy and pregnancy. All participants were residents of Pomeranian region of Poland and were exposed to the same environmental factors. Thus, there were no differences between the subjects in exposition to the pollution that could affect serum levels of measured cytokines.

Fasting blood samples were drawn from all the participants in sitting position from forearm vein and subsequently were used to measure standard clinical parameters (Table 1 and Table 2) and serum concentration of endostatin, VEGF, IL-8, angiogenin, and bFGF.

**Table 2:** Characteristics of the plasma lipidogram.

Parameter	Hypertensive patients			Healthy individuals		
	Normal values (n)	Outside reference range (n)	Proportion normal/outside reference range	Normal values (n)	Outside reference range (n)	Proportion normal/outside reference range
TCh	23	59	0.38	7	27	0.25
LDL	25	57	0.43	8	26	0.30
HDL	66	16	4.12	31	3	10.33
TG	62	20	3.1	27	7	3.85

TCh = total cholesterol, LDL = low-density lipoprotein, HDL = high-density lipoprotein,

TG = triglicerides

The study was conducted in accordance with the Declaration of Helsinki principles and was approved by the Ethics Committee of the Medical University of Gdańsk, Poland. Written informed consent was received from all the participants.

### Sample preparation

Serum was obtained from blood samples after centrifugation at 500xg for 15 minutes. Then serum was apportioned into 0.5 ml aliquots, and stored at -80°C until analysis.

### Analysis of concentration of selected mediators affecting angiogenesis

Angiogenin and endostatin concentration were measured with ELISA Quantikine^®^ tests (R&D Systems, USA) according to the manufacturer instructions. The tests’ sensitivities were 6 pg/ml and 0.063 ng/ml, respectively. VEGF, IL-8, and bFGF were analyzed with flow cytometer (Canto II; BD Biosciences, USA) using Cytometric Bead Array (CBA) Human Soluble Protein Flex Sets (BD Biosciences, USA). The test sensitivities for measured proteins were as follow: 4.5 pg/ml, 1.2 pg/ml, and 3.4 pg/ml (detailed description of the method in S1 Appendix and S1 Fig).

Both methods, CBA and ELISA interpolate concentrations of the measured cytokines from the standard curves, quantifying their levels in analyzed samples and provide comparable sensitivity and specificity [34–36].

The main advantage of CBA technique in comparison with traditional ELISA is low sample volume required and low time to results ratio, as multiple cytokines can be measured in 1 tube simultaneously. Nevertheless, endostatin and angiogenin were measured with conventional ELISA, because they are present in human serum in significantly higher concentrations than VEGF, IL-8 and bFGF. Measurement of serum endostatin and angiogenin concentrations requires multiple dilutions of the samples that makes VEGF, IL-8 and bFGF undetectable, as they are present in serum in about 1000 fold lower levels than endostatin and angiogenin.

### Statistical analysis

The results were calculated with Statistica 10.0 software (StatSoft, Poland). As indicated by the distribution of the variables, data were analyzed with Mann-Whitney U test and Spearman’s rank correlation for nonparametric data. In addition, multiple regression analyses were performed to verify if age, body mass index (BMI) and serum lipids affect levels of studied cytokines. Values of *p*<0.05 were considered statistically significant.

## Results

### Hypertension is associated with increased serum concentration of endostatin, VEGF and IL-8

**Fig 1:**
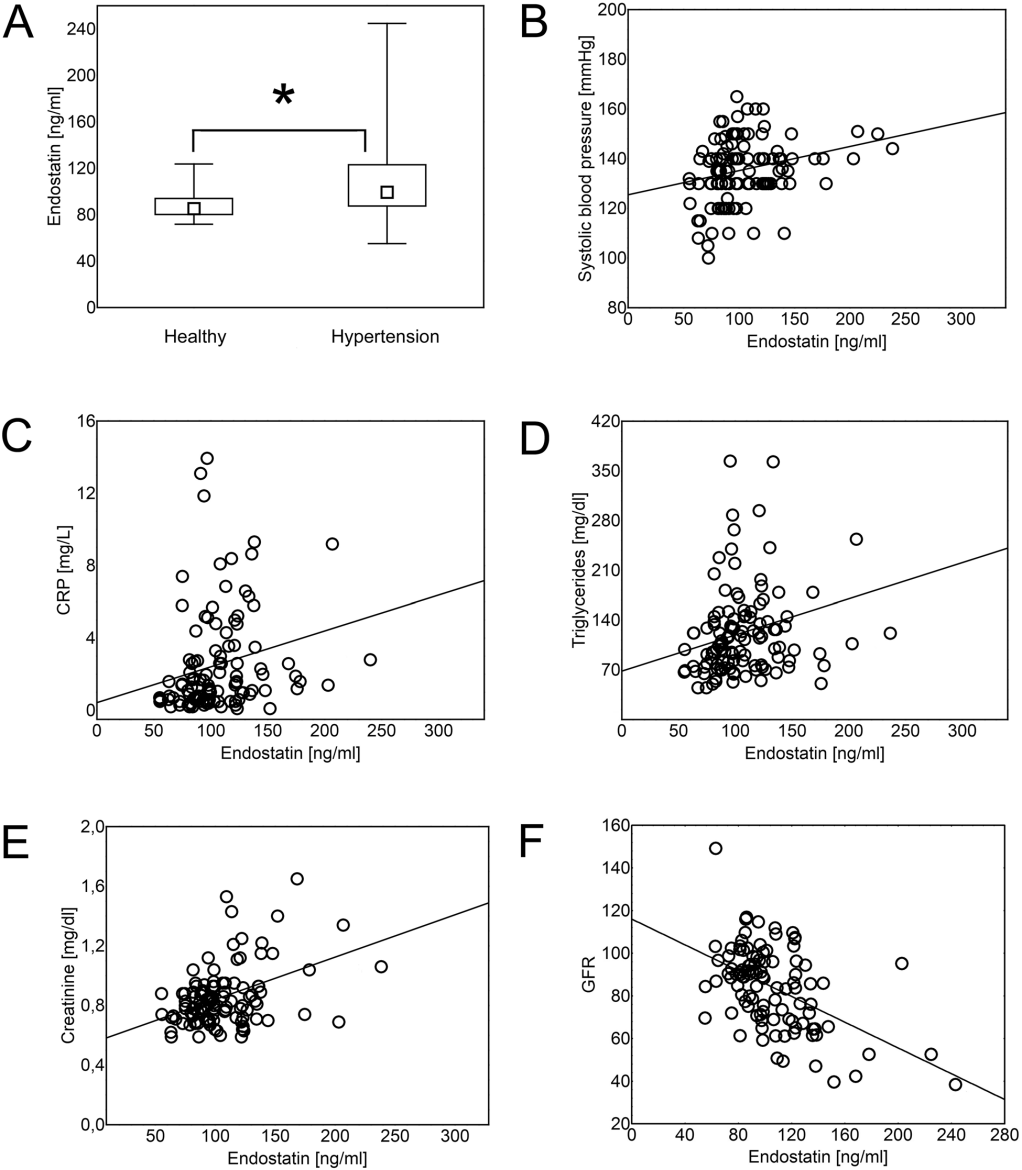
Endostatin concentration in hypertension. Hypertension was associated with increased serum levels of endostatin (Mann- Whitney U test; p = 8x10^-4^). The data are presented as medians (symbols inside the boxes), 25–75% percentiles (boundaries of the boxes) and minimum—maximum (error bars outside the boxes). Statistical significance (p<0.05) is marked with “*” (A). Endostatin concentration was in positive correlation (Spearman’s rank correlation) with systolic blood pressure (B; R = 0.287, p = 1x10^-3^), CRP (C; R = 0,326,p = 4x10^-4^), triglycerides (D; R = 0.260, p = 4x10^-3^), serum creatinine (E; R = 0,313 p = 8x10^-4^) and in negative correlation with glomerular filtration rate (GFR) (F; R = -0.42, p = 1x10^-5^). Correlations were calculated for both- hypertensive and healthy individuals together.

**Fig 2:**
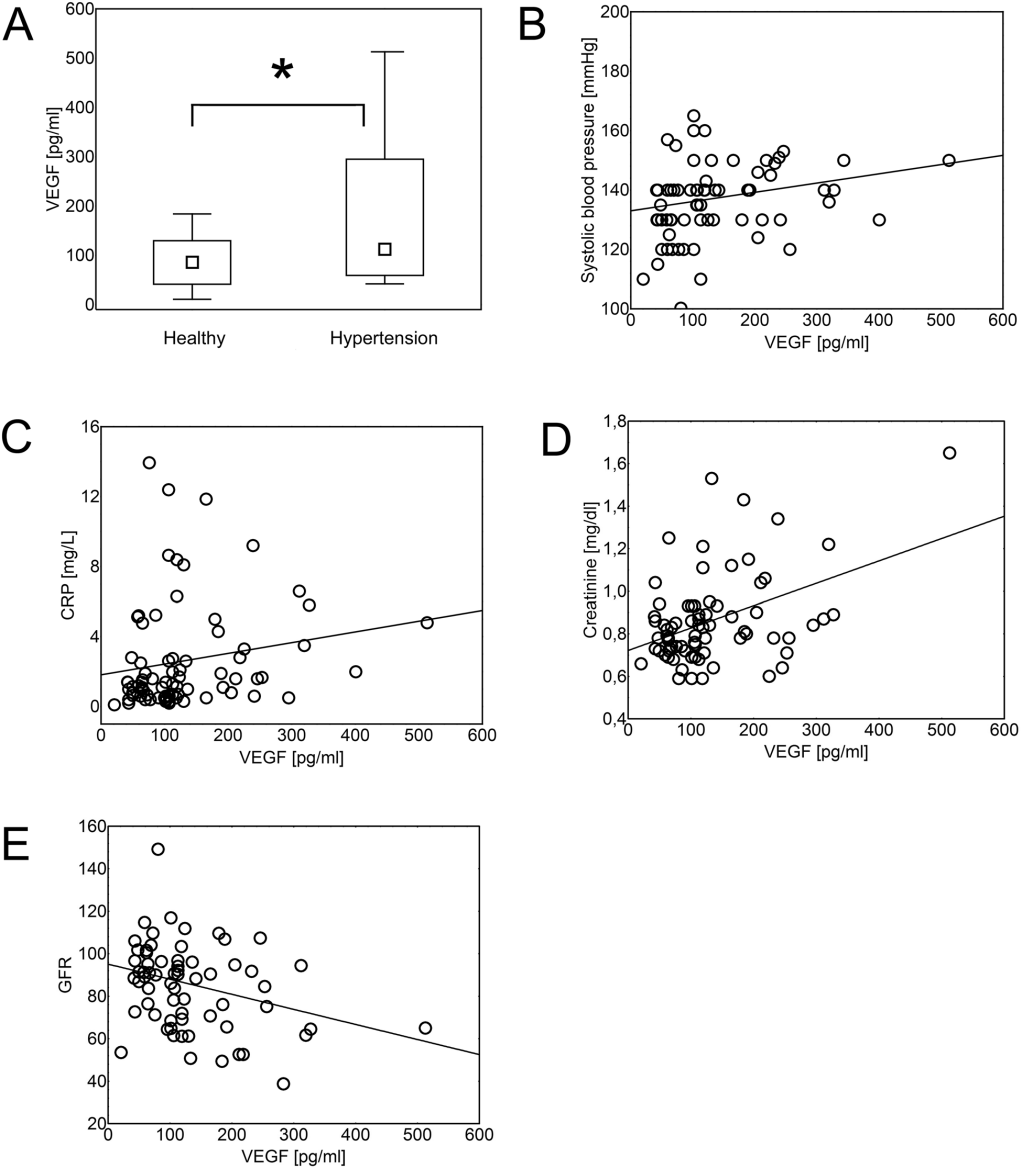
VEGF concentration in hypertension. Hypertension was associated with increased serum levels of VEGF (Mann- Whitney U test; p = 0.02). The data are presented as medians (symbols inside the boxes), 25–75% percentiles (boundaries of the boxes) and minimum—maximum (error bars outside the boxes). Statistical significance (p<0.05) is marked with “*” (A). Concentration of VEGF was in positive correlation (Spearman’s rank correlation) with systolic blood pressure (B; R = 0,337, p = 3x10^-3^), CRP (C; R = 0.265, p = 0.02), serum creatinine (D; R = 0.253, p = 0.03) and in negative correlation with glomerular filtration rate (GFR) (E; R = -0.24, p = 0.04). Correlations were calculated for both- hypertensive and healthy individuals together.

**Fig 3:**
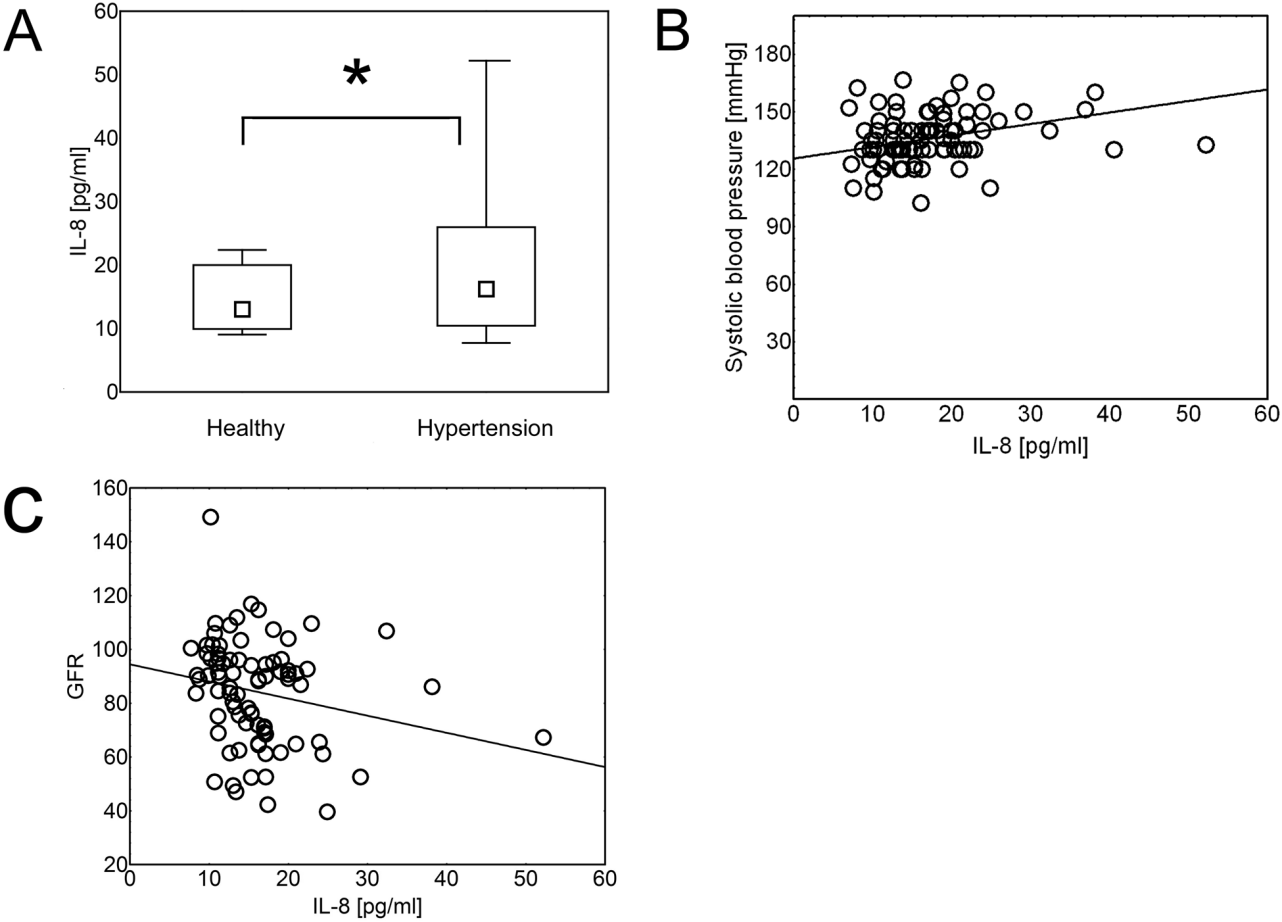
IL-8 concentration in hypertension. Hypertension was associated with increased serum levels of IL = 8 (Mann- Whitney U test; p = 0.02). The data are presented as medians (symbols inside the boxes), 25–75% percentiles (boundaries of the boxes) and minimum—maximum (error bars outside the boxes). Statistical significance (p<0.05) is marked with “*” (A). Concentration of IL = 8 was in positive correlation (Spearman’s rank correlation) with systolic blood pressure (B; R = 0,326, p = 1x10^-3^) and in negative correlation with glomerular filtration rate (GFR) (C; R = -0.26, p = 0.01). Correlations were calculated for both- hypertensive and healthy individuals together.

Analysis showed that patients with hypertension were characterized by higher serum levels of endostatin (Fig 1), VEGF (Fig 2) and IL-8 (Fig 3), than healthy individuals (p = 8x10^-4^, p = 0.02, and p = 0.02, respectively). The same differences were found when the data were re-analyzed in order to express the amount of the cytokine of interest in relation to the amount of total serum protein for each evaluated sample (S2 Fig, p = 0.047, p = 0.021, and p = 0.014, respectively). Serum concentration of theses cytokines was in positive correlation with systolic blood pressure (R = 0.287, p = 1x10^-3^; R = 0,337, p = 3x10^-3^; and R = 0,326, p = 1x10^-3^, respectively; Figs 1–3) and in negative correlation with glomerular filtration rate (GFR) (R = -0.42, p = 1x10^-5^; R = -0.24, p = 0.04; and R = -0.26, p = 0.01, respectively; Figs 1–3).

In addition, concentration of endostatin correlated positively with level of C-reactive protein (CRP; R = 0,326,p = 4x10^-4^), triglycerides (R = 0.260, p = 4x10^-3^) and serum creatinine (R = 0,313 p = 8x10^-4^), while serum VEGF levels were in positive correlation with CRP (R = 0.265, p = 0.02) and serum creatinine concentration (R = 0.253, p = 0.03; Fig 2).

**Fig 4:**
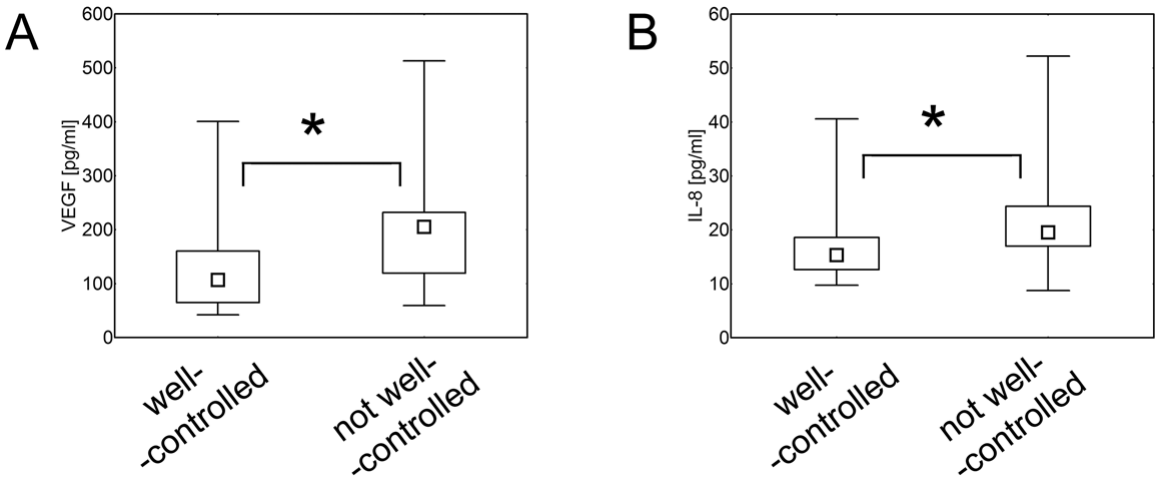
Control of hypertension and serum concentration of cytokines. Patients with well controlled hypertension had lower serum levels of VEGF (A) and IL = 8 (B) (Mann- Whitney U test; p = 8x10^-3^ and p = 7x10^-3^, respectively) in comparison with individuals with not well-controlled hypertension. The data are presented as medians (symbols inside the boxes), 25–75% percentiles (boundaries of the boxes) and minimum—maximum (error bars outside the boxes). Statistical significance (p<0.05) is marked with “*”.

When hypertensive patients were divided into well-controlled (systolic blood pressure <140 mmHg and diastolic blood pressure <90 mmHg) and not well-controlled (systolic blood pressure ≥140 mmHg and/or diastolic blood pressure ≥90 mmHg), it was found that individuals who were not well controlled by the therapy- had higher serum levels of VEGF and IL-8 (p = 8x10^-3^ and p = 7x10^-3^, respectively), than patients with well-controlled hypertension (Fig 4).

### Hypertensive patients are characterized by decreased serum levels of angiogenin and bFGF

**Fig 5:**
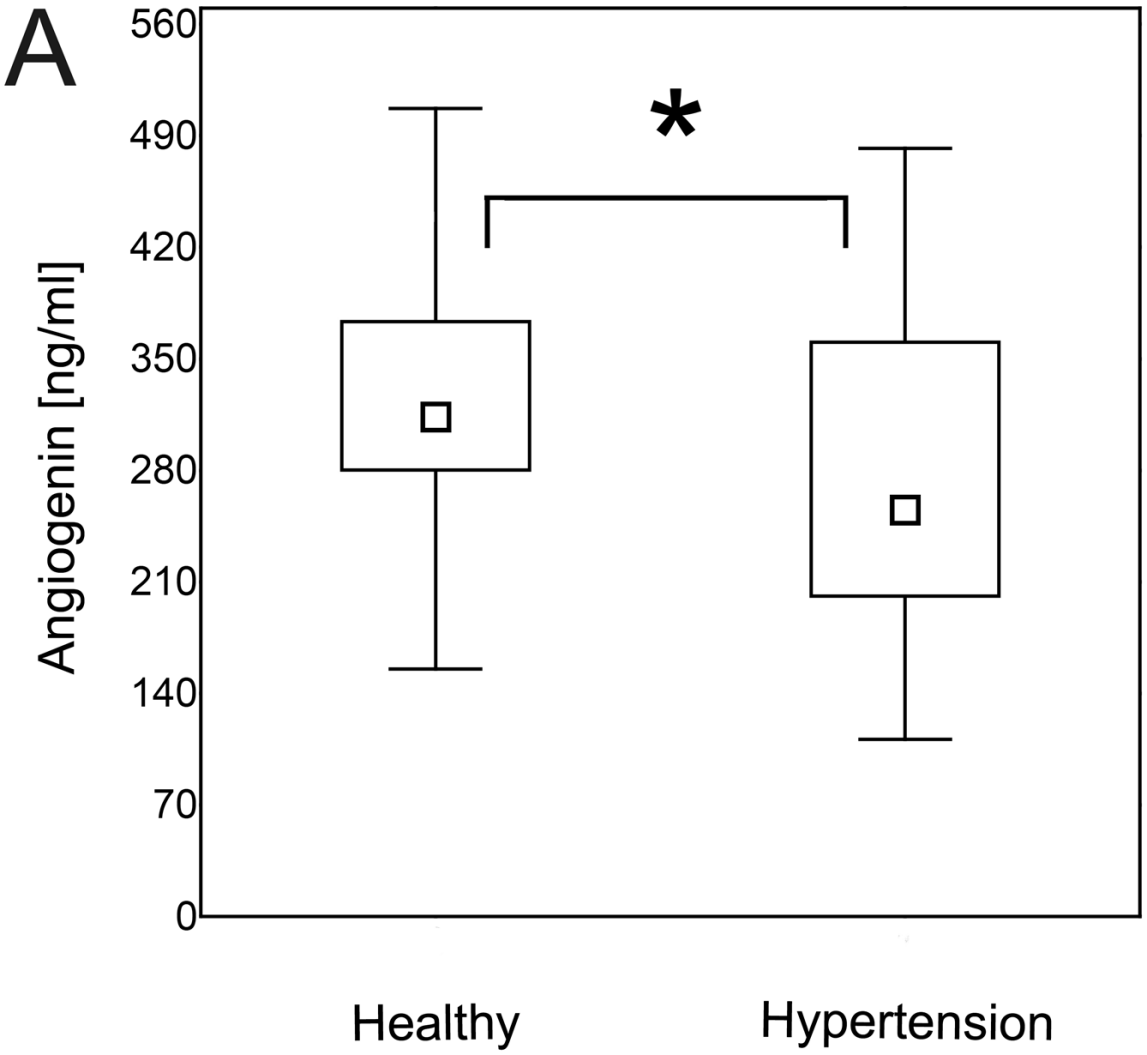
Angiogenin concentration in hypertension. Patients with hypertension were characterized by lower serum concentration of angiogenin than healthy individuals (Mann- Whitney U test; p = 0.01). The data are presented as medians (symbols inside the boxes), 25–75% percentiles (boundaries of the boxes) and minimum—maximum (error bars outside the boxes). Statistical significance (p<0.05) is marked with “*”.

**Fig 6:**
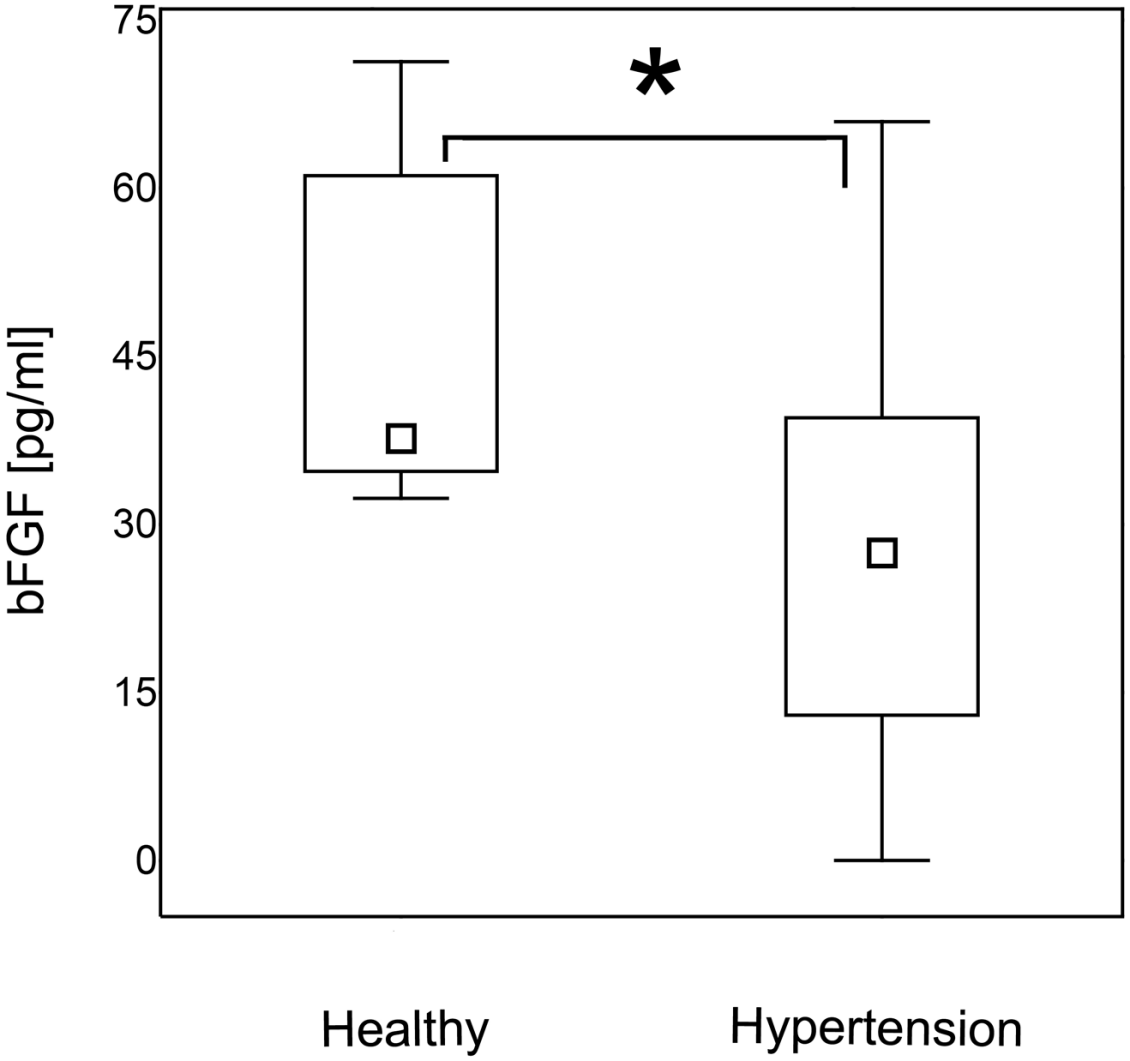
bFGF concentration in hypertension. Patients with hypertension were characterized by lower serum concentration of bFGF than healthy individuals (Mann- Whitney U test; p = 0.01). The data are presented as medians (symbols inside the boxes), 25–75% percentiles (boundaries of the boxes) and minimum—maximum (error bars outside the boxes). Statistical significance (p<0.05) is marked with “*”.

Patients with hypertension had significantly lower serum levels of angiogenin (Fig 5) and bFGF (Fig 6) than normotensive controls (p = 0.01 for both). The same differences were found when the data were re-analyzed in order to express the amount of the cytokine of interest in relation to the amount of total serum protein for each evaluated sample (S2 Fig, p = 0.043 and p = 0.040, respectively).

### Obesity does not affect endostatin, VEGF, IL-8, angiogenin and bFGF levels in serum of hypertensive and healthy individuals

As previous studies suggested that production of certain cytokines can be affected by age, obesity and serum lipids [37–41] we aimed to verify if these factors affect also endostatin, VEGF, IL-8, angiogenin and bFGF measured in our study. The best fitting models of multiple regression analyses with significant independent predictors such as: age, BMI, triglycerides (TG) and low-density lipoproteins (LDL) showed no association between these factors and studied cytokines. There were only two exceptions for endostatin concentration (S1 Table, S2 Table, S3 Table, S4 Table and S5 Table), that was found to be affected by patient’s age and serum TG levels (S5 Table; β = 0,379, p = 4x10^-4^ and β = 0,319, p = 0.001, respectively).

When all subjects were divided into subsequent 3 groups: 1). normal BMI (<25; n = 58), 2). overweight (25–29.99; n = 41) and 3). obese (≥30; n = 17), no statistically significant differences between the groups in terms of serum cytokine levels were found (data not shown).

### Statin treatment is associated with higher serum levels of VEGF

**Fig 7:**
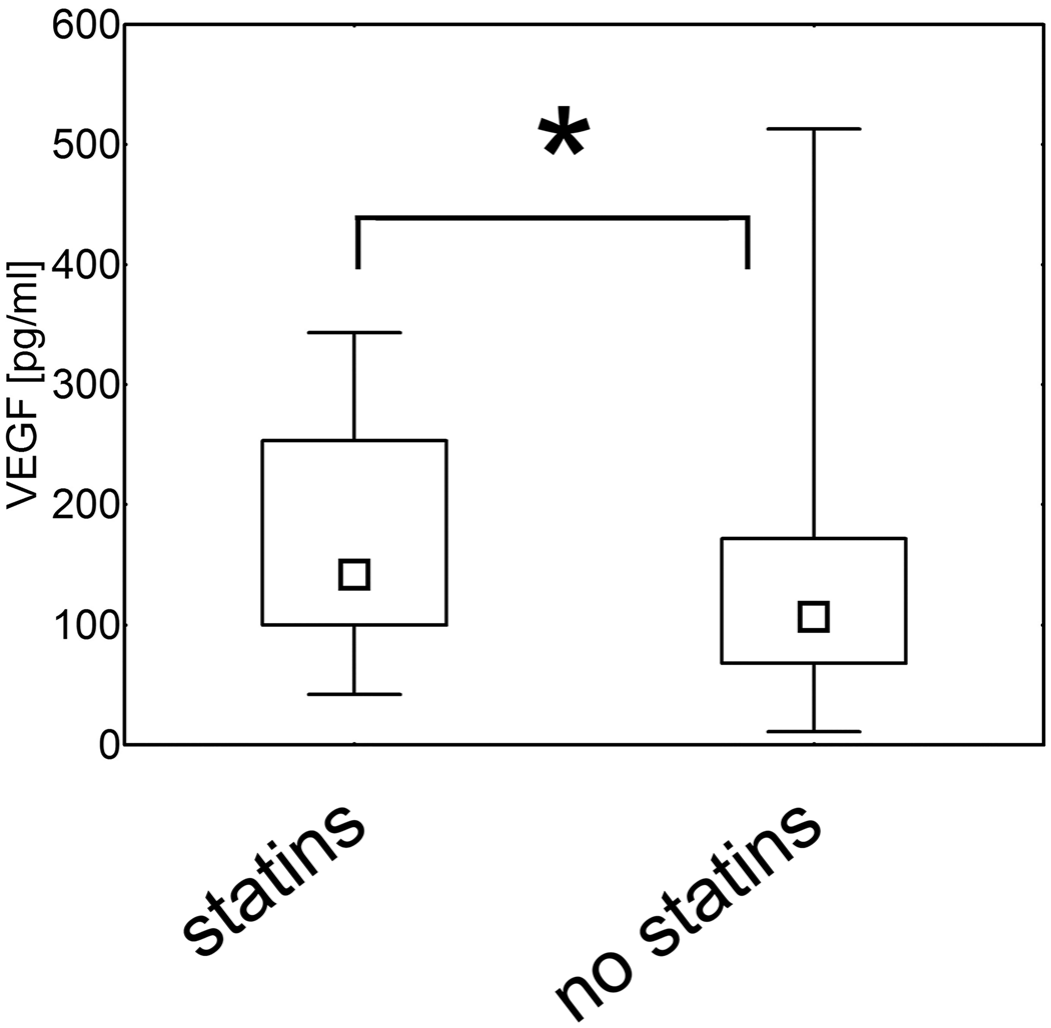
Statin treatment and serum levels of VEGF. Healthy and hypertensive individuals treated with statins had increased serum levels of VEGF when compared with not treated subjects (Mann- Whitney U test; p = 0.04). The data are presented as medians (symbols inside the boxes), 25–75% percentiles (boundaries of the boxes) and minimum-maximum (error bars outside the boxes). Statistical significance (p<0.05) is marked with “*”.

In hypertensive and control group 12.1% and 14.7% of the patients were treated with statins, respectively (Table 1). As previous studies reported that the drugs may affect production of cytokines [42], we aimed to verify if and how these medicaments influence levels of studied mediators in our patients. We found that individuals who were taking statins had higher serum concentrations of VEGF than non-treated participants (p = 0.04; Fig 7). No differences between these two groups were found for serum levels of endostatin, IL-8, angiogenin and bFGF (p = 0.57, p = 0.43, p = 0.36 and p = 0.43, respectively; data not shown).

## Discussion

The current study shows that arterial hypertension is characterized by imbalance of pro-angiogenic versus anti-angiogenic factors.

In our study hypertensive patients were characterized by increased levels of endostatin that is a potent anti-angiogenic factor [18–21] and decreased serum concentration of pro-angiogenic angiogenin and bFGF [12–17,20, 43]. These observations support the hypothesis that hypertension is associated with impaired angiogenesis [4,12,44–45]. Enhanced production of anti-angiogenic factors and concomitant decreased synthesis of pro-angiogenic mediators may lead to microvascular rerefaction that causes increase in peripheral resistance and thus leads to hypertension [4,12].

Physiologically serum concentration of mediators that regulate angiogenesis is maintained in defined range. Nevertheless, multiple factors may affect their production, leading to acute and/or chronic disorders. It was suggested previously that serum lipids and obesity may change concentration of several cytokines [38–41], therefore we aimed to verify if endostatin, VEGF, IL-8, angiogenin and bFGF are affected by these factors in our patients. Despite we divided all participants according to their BMI into 3 groups (normal BMI, overweight and obese), we did not find differences between the groups in terms of serum concentrations of studied cytokines. Multiple regression analyses with significant independent predictors such as: age, BMI, TG and LDL showed no association between these factors and VEGF, IL-8, angiogenin or bFGF. Among studied cytokines only endostatin concentration was found to be affected by patient’s age and serum levels of TG. Nevertheless, healthy and hypertensive individuals were matched for age, and proportions of the subjects with normal and elevated levels of TG in both groups were similar. Therefore, influence of age and TG on endostatin levels was comparable in healthy and hypertensive individuals. These data indicate that differences between the two groups in serum concentrations of all studied cytokines resulted neither from various age, serum lipid levels, nor BMI values, but they were related with presence or absence of hypertension.

As it was mentioned before, patients with hypertension were characterized by lower serum levels of angiogenin. Noteworthy, angiogenin is not only a protein that stimulates angiogenesis [13–17], but also exhibits anti-inflammatory and immunosuppressive activity [17]. Therefore, angiogenin not only stimulates development of new vessels but also protects already existing vessels from damaging impact of proinflammatory factors. In addition, previous studies reported its cytoprotectiv activity [46–47]. It was also suggested that angiogenin triggers integrated stress response program and serves as a stress-induced mediator acting in paracrine mode to protect neighboring cells from deleterious impact of stress [46]. It was also shown that angiogenin binds actin on the surface of endothelial cells and the complex angiogenin-actin stimulates tissue plasminogen activator (t-PA) to produce plasmin from plasminogen [48–49]. These observations indicate that angiogenin is an anti-thrombotic factor. Therefore, decreased level of angiogenin in hypertension may have severe clinical consequences. Hypertension is the major risk factor for thrombotic events [50–51] and low serum level of anti-thrombotic angiogenin may play an important role in their onset. In addition, it was found that angiogenin concentration increases physiologically in response to acute phase proteins [48]. Lower concentration of this mediator in our hypertensive patients despite elevated CRP levels in this group may suggest that this mechanism is impaired in hypertension. Previously, angiogenin was only measured in pregnancy induced hypertension [52] and hypertensive patients with heart failure [53], while there were no data regarding its serum levels in arterial hypertension without cardiac complications as we present in the current study. Therefore, bearing in mind all mentioned properties of angiogenin, our observations shed new light on the pathogenesis of hypertension and development of its complications.

In addition, in our study hypertensive patients had increased serum concentration of VEGF that is a proangiogenic factor closely connected with inflammation and IL-8 that is another inflammatory mediator [13,23–27]. One of the most important factors that induce expression of VEGF is hypoxia that can result from vessel damage. In addition, it was reported that VEGF released from activated platelets at the side of vessel wall damage attracts circulating neutrophils and monocytes that are crucial for induction of inflammatory response [54]. Moreover, VEGF was shown to play an important role in induction of inflammatory process in autoimmune diseases and their complications not only via altered angiogenesis but also due to its proinflammatory activity [13,23,55]. Inter alia VEGF was found to increase the production of crucial inflammatory mediators such as tumor necrosis factor α (TNF-α), IL-6, IFN-γ and inhibit production of anti-inflammatory IL-10 by human and animal peripheral blood mononuclear cells (PBMC) and thus lead to chronic disease exacerbation [25,56]. Similar to VEGF, IL-8 is released in response to any injury of endothelium. This may explain increased levels of these chemokines in serum of our patients. Noteworthy, when we divided hypertensive individuals into well- and not well-controlled by the hypotensive therapy, we found that uncontrolled patients had significantly elevated serum concentrations of VEGF and IL-8. In addition, IL-8 prestored in Weibel-Palade bodies of endothelial cells can be released after stimulation with other inflammatory mediators such as IL-1 or TNF-α [57]. Recently, Martin et al. showed that IL-8 up-regulates synthesis of VEGF (in nuclear factor κB dependent mode; NFκB) in endothelial cells [58]. In our study serum concentration of VEGF was in positive correlation with CRP levels. These observations support the hypothesis that increase in serum VEGF levels in the studied hypertensive patients reflects response to endothelium injury and might be a part of proinflammatory response. Noteworthy, previous studies on diseases with inflammatory component showed that increased levels of VEGF are rather associated with abnormal angiogenesis and endothelial dysfunction, than with beneficial vessel regeneration [59].

In addition, in our study VEGF and IL-8 correlated negatively with glomerular filtration rate (GFR) and VEGF was also in positive correlation with serum creatinine concentration. These observations suggest that increase of serum VEGF concentration may be an early marker of kidney impairment that is one of the most common complications of hypertension [10,60]. These data correspond to the previous studies on diabetes, where increased serum and urine concentration of VEGF was found to be an early marker of nephropathy and a likely contributory factor for diabetic kidney disease [30]. Recent studies also suggest that the negative effects of VEGF are amplified in the settings of endothelial dysfunction and low nitric oxide (NO) levels, which are a common feature of hypertension. It was observed that at low levels of NO, inflammatory properties of VEGF are amplified (strong activation of macrophages), may lead to dysregulation of the vasculature, and thus “uncoupling” of the VEGF-NO axis may contribute to the pathology of the kidney [30].

In our study statin treated individuals had higher serum levels of VEGF, suggesting that statins stimulate VEGF production. Nevertheless, despite the percentage of statin treated individuals was higher in the control group, healthy individuals had lower serum concentration of VEGF than hypertensive patients. This indicates that statin treatment did not obscure the results and suggests that if all studied subjects were not on statin therapy, the differences in VEGF levels between healthy and hypertensive subjects would be even more pronounced. These observations underline the significance and utility of VEGF as a marker of hypertension and potential predictor of its complications. In addition, these data shed new light on consequences of statin therapy. For several years this group of drugs was considered to have beneficial anti-inflammatory impact [42, 61–62]. Nevertheless, the most recent data do not confirm these observations [63–64] and even suggest that statins may predispose to cancer development/recurrence [65–66]. Our data are in accordance with these recent reports and seems to partially explain why statins may be involved in carcinogenesis. In our study individuals treated with statins had increased serum levels of VEGF that is a known mediator of tumor growth and metastases, while anti-VEGF drugs have been used for years as effective therapy of several cancers [67–70]. According to our knowledge our study is the first suggesting that statins may increase production of VEGF, but can be supported by observations of Llevadot and Asahara who found that statins promote angiogenesis and vasculogenesis in a way similar to VEGF [71].

In summary, in the present study we found that hypertensive patients had higher serum levels of endostatin that is a potent angiogensis inhibitor and lower serum concentration of pro-angiogenic bFGF and angiogenin than healthy individuals. As angiogenin protects blood vessels from negative effects of inflammation, has cytoprotective potential, is an important inducer of new blood vessel formation and is required for pro-angiogenic activity of other angiogenesis stimulators, its decreased production in hypertensive patients may play an important role in pathogenesis of hypertension and its complications. Concomitantly, hypertension was accompanied by increased serum concentration of IL-8 and VEGF that reflects inflammatory response and abnormal angiogenesis. The phenomenon was the most pronounced in uncontrolled hypertension. These data all together show that qualitative and quantitative changes in pro-angiogenic and anti-angiogenic factors in the background of inflammation are the hallmarks of arterial hypertension.

## Supporting Information

S1 Appendix Detailed description of measurement of VEGF, IL-8 and bFGF levels with flow cytometry. The file describes the Cytometric Bead Array (CBA) technique used for measurement of serum VEGF, IL-8, and bFGF levels.
(DOC)

S1 Fig Cytometric measurement of VEGF, IL-8 and bFGF. Figure briefly describes the method of cytometric measurement of serum concentration of VEGF, IL-8 and bFGF with Cytometric Bead Array (CBA). A) FSC-A vs SSC-A dot plot shows a mix of three types of beads used for the measurement of VEGF, IL-8 and bFGF. Cell conglomerates (visible as black dots) are excluded from the analysis gate. B) Dot plot visualizes position of each group of beads on APC-A and APC-Cy7-A axes. Each bead set has different alphanumeric position on the dot-plot. C-D) APC-A vs PE-A dot plots show exemplary results obtained for 2 different samples. Various concentrations of VEGF, IL-8, and bFGF are visualized on PE-A axis. The higher fluorescence intensity of PE detection reagent, the higher concentration of the analyte.
(TIF)

S2 Fig Serum concentration of endostatin, VEGF, IL-8, angiogenin and bFGF in relation to the amount of total serum protein. Serum concentrations of endostatin, VEGF, IL-8, angiogenin and bFGF were re-analyzed in order to express the amount of each cytokine in relation to the total serum protein level. After this recalculation hypertensive patients had still higher serum levels of endostatin (A; p = 0.047), VEGF(B; p = 0.021), and IL-8, (C; p = 0.014), and lower serum concentration of angiogenin (D; p = 0.043) and bFGF (E; p = 0.040) The data were calculated with Mann- Whitney U test and are presented as medians (symbols inside the boxes), 25–75% percentiles (boundaries of the boxes) and minimum—maximum (error bars outside the boxes). Statistical significance (p<0.05) is marked with “*”
(TIF)

S1 Table Assessment of the impact of age, BMI and serum lipid levels on serum VEGF concentration. Multiple regression analysis was used to assess the influence of independent predictors such as: age, BMI, triglycerides and LDL on serum levels of VEGF.
(DOC)

S2 Table Assessment of the impact of age, BMI and serum lipid levels on serum IL-8 concentration. Multiple regression analysis was used to assess the influence of independent predictors such as: age, BMI, triglycerides and LDL on serum levels of IL-8.
(DOC)

S3 Table Assessment of the impact of age, BMI and serum lipid levels on serum angiogenin concentration. Multiple regression analysis was used to assess the influence of independent predictors such as: age, BMI, triglycerides and LDL on serum levels of angiogenin.
(DOC)

S4 Table Assessment of the impact of age, BMI and serum lipid levels on serum bFGF concentration. Multiple regression analysis was used to assess the influence of independent predictors such as: age, BMI, triglycerides and LDL on serum levels of bFGF.
(DOC)

S5 Table Assessment of the impact of age, BMI and serum lipid levels on serum endostatin concentration. Multiple regression analysis was used to assess the influence of independent predictors such as: age, BMI, triglycerides and LDL on serum levels of endostatin.
(DOC)
