# Supplementary material for: Arterial Hypertension Is Characterized by Imbalance of Pro-Angiogenic versus Anti-Angiogenic Factors
Source: PLoS One. 2015 May 7;10(5):e0126190. doi: 10.1371/journal.pone.0126190 (PMC4423857; doi:10.1371/journal.pone.0126190)
Supplement: S1 Appendix — The file describes the Cytometric Bead Array (CBA) technique used for measurement of serum VEGF, IL-8, and bFGF levels. (DOC) [file pone.0126190.s001.doc]

**S1 Appendix. Detailed description of measurement of VEGF, IL-8 and bFGF levels with flow cytometry**

The file describes the Cytometric Bead Array (CBA) technique used for measurement of serum VEGF, IL-8, and bFGF levels.

VEGF, IL-8, and bFGF were analyzed with flow cytometer (Canto II; BD Biosciences, USA) using Cytometric Bead Array (CBA) Human Soluble Protein Flex Sets (BD Biosciences, USA). The method combines the idea of standard ELISA test with flow cytometry and thus enables simultaneous analysis of few mediators in one sample at one time with low sample volume requirement. In the test we used a mix of three types of beads (Fig.S1A) coated with antibodies specific for VEGF, IL-8 or bFGF that enabled detection of these cytokines. Each bead set had different fluorescence intensity of allophycocyanin (APC) and allophycocyanin-Cy7 (APC-Cy7), resulting in different alphanumeric position at the dot-plot and clear separation of the signals from all cytokines (Fig.S1B). Subsequently, antibodies with the same specificity (against VEGF, IL-8, and bFGF) but conjugated with phycoerythrin (PE) were added. These antibodies are called PE detection reagent and serve for quantitative measurement of the analyzed mediators. The higher fluorescence intensity of PE, the higher concentration of the analyte (Fig.S1C-D). In addition, each set for analysis of each mediator comprised standard for preparation of standard solutions and generation of the standard curve. Thus in CBA method we interpolated concentrations of the measured cytokines from the standard curves that is a common way of quantifying the concentration of a sample used also in standard ELISA.

In summery, due to various fluorescence intensities of APC, APC-Cy7 and PE of used reagents we were able to detect the separate signal from each cytokine and measure the cytokine concentrations simultaneously. Then, the data were analyzed using the FCAP Array Software (BD, USA).
