# Supplementary figures and images for: Arterial Hypertension Is Characterized by Imbalance of Pro-Angiogenic versus Anti-Angiogenic Factors
Source: PLoS One. 2015 May 7;10(5):e0126190. doi: 10.1371/journal.pone.0126190 (PMC4423857; doi:10.1371/journal.pone.0126190)

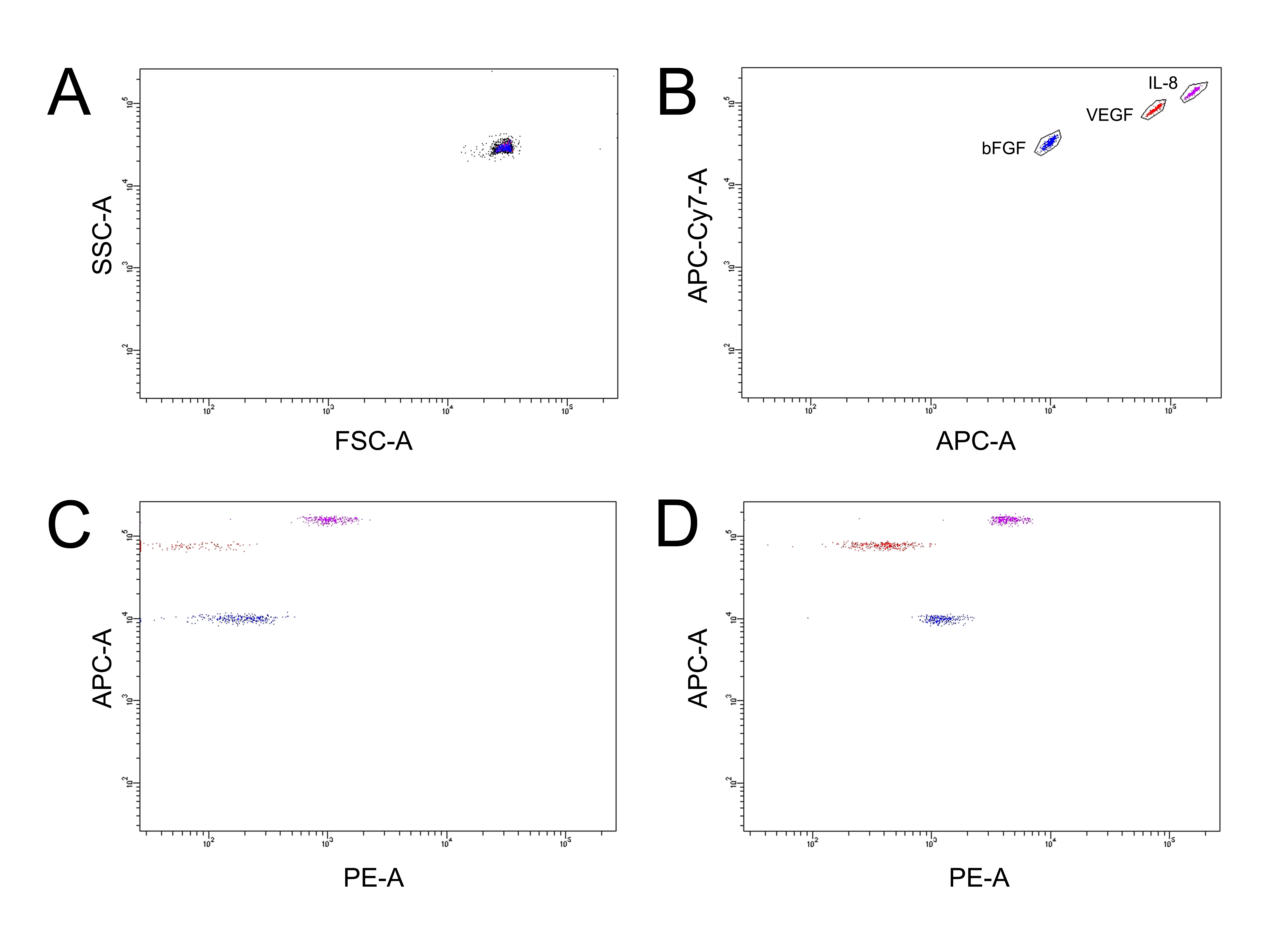

Supplement: S1 Fig — Figure briefly describes the method of cytometric measurement of serum concentration of VEGF, IL-8 and bFGF with Cytometric Bead Array (CBA). A) FSC-A vs SSC-A dot plot shows a mix of three types of beads used for the measurement of VEGF, IL-8 and bFGF. Cell conglomerates (visible as black dots) are excluded from the analysis gate. B) Dot plot visualizes position of each group of beads on APC-A and APC-Cy7-A axes. Each bead set has different alphanumeric position on the dot-plot. C-D) APC-A vs PE-A dot plots show exemplary results obtained for 2 different samples. Various concentrations of VEGF, IL-8, and bFGF are visualized on PE-A axis. The higher fluorescence intensity of PE detection reagent, the higher concentration of the analyte. (TIF) [file pone.0126190.s002.tif]

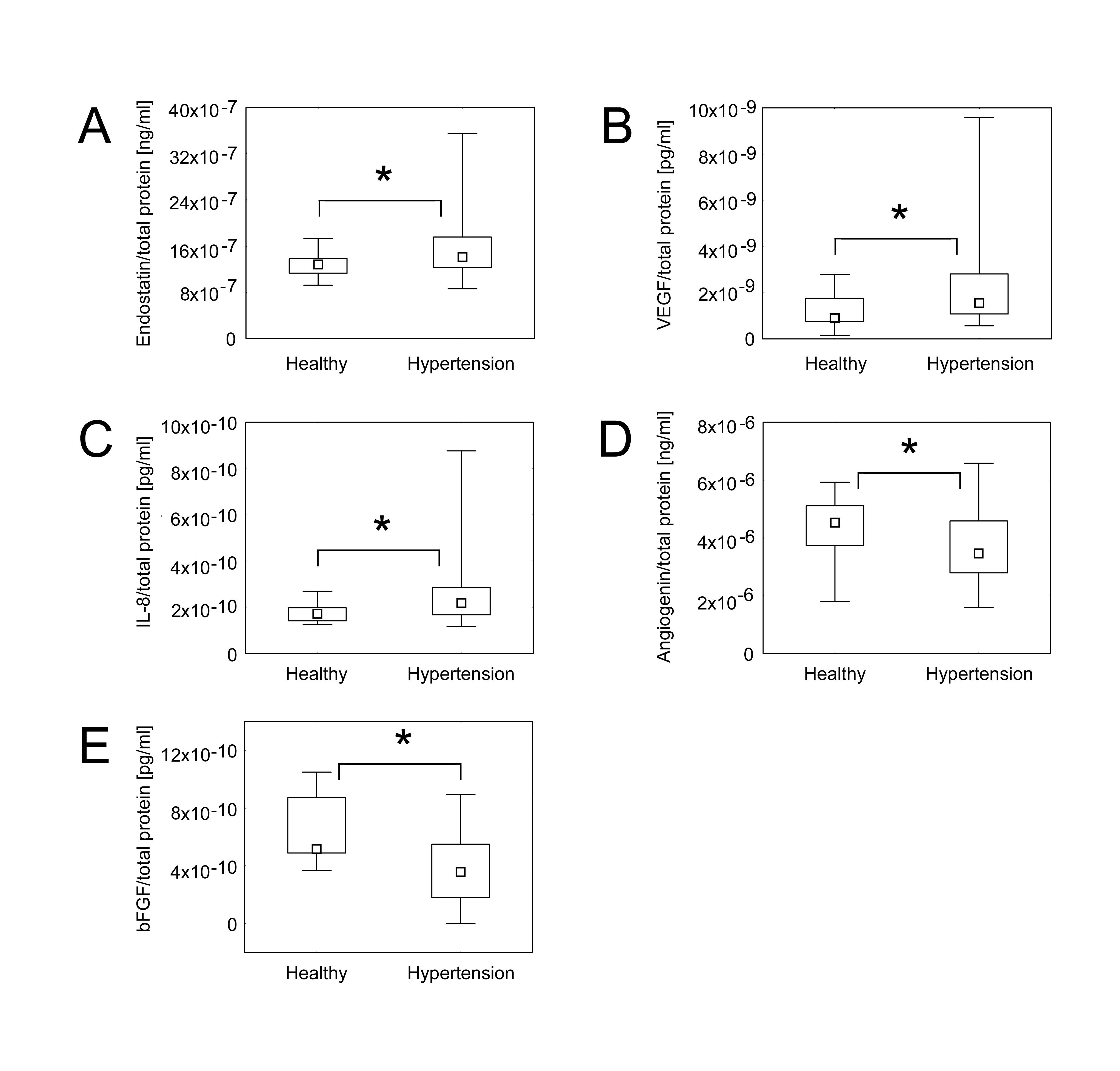

Supplement: S2 Fig — Serum concentrations of endostatin, VEGF, IL-8, angiogenin and bFGF were re-analyzed in order to express the amount of each cytokine in relation to the total serum protein level. After this recalculation hypertensive patients had still higher serum levels of endostatin (A; p = 0.047), VEGF(B; p = 0.021), and IL-8, (C; p = 0.014), and lower serum concentration of angiogenin (D; p = 0.043) and bFGF (E; p = 0.040) The data were calculated with Mann- Whitney U test and are presented as medians (symbols inside the boxes), 25–75% percentiles (boundaries of the boxes) and minimum—maximum (error bars outside the boxes). Statistical significance (p<0.05) is marked with “*” (TIF) [file pone.0126190.s003.tif]
