# Supplementary material for: Arterial Hypertension Is Characterized by Imbalance of Pro-Angiogenic versus Anti-Angiogenic Factors
Source: PLoS One. 2015 May 7;10(5):e0126190. doi: 10.1371/journal.pone.0126190 (PMC4423857; doi:10.1371/journal.pone.0126190)
Supplement: S1 Table — Multiple regression analysis was used to assess the influence of independent predictors such as: age, BMI, triglycerides and LDL on serum levels of VEGF. (DOC) [file pone.0126190.s004.doc]

**S1 Table. Assessment of the impact of age, BMI and serum lipid levels on serum VEGF concentration**

Multiple regression analysis was used to assess the influence of independent predictors such as: age, BMI, triglycerides and LDL on serum levels of VEGF.

| Variable | *β* | *P value* |
| --- | --- | --- |
| Age | 0,150 | 0,222 |
| BMI | 0,144 | 0,268 |
| TG | 0,015 | 0,908 |
| LDL | 0,026 | 0,838 |

BMI=body mass index, TG=triglycerides, LDL=low-density lipoprotein
