# Supplementary material for: Arterial Hypertension Is Characterized by Imbalance of Pro-Angiogenic versus Anti-Angiogenic Factors
Source: PLoS One. 2015 May 7;10(5):e0126190. doi: 10.1371/journal.pone.0126190 (PMC4423857; doi:10.1371/journal.pone.0126190)
Supplement: S2 Table — Multiple regression analysis was used to assess the influence of independent predictors such as: age, BMI, triglycerides and LDL on serum levels of IL-8. (DOC) [file pone.0126190.s005.doc]

**S2 Table. Assessment of the impact of age, BMI and serum lipid levels on serum IL-8 concentration**

**Multiple regression analysis was used to assess the influence of independent predictors such as: age, BMI, triglycerides and LDL on serum levels of IL-8.**

| Variable | *β* | *P value* |
| --- | --- | --- |
| Age | 0,155 | 0,147 |
| BMI | -0,007 | 0,950 |
| TG | 0,008 | 0,945 |
| LDL | 0,190 | 0,087 |

BMI=body mass index, TG=triglycerides, LDL=low-density lipoprotein
