# Supplementary material for: Arterial Hypertension Is Characterized by Imbalance of Pro-Angiogenic versus Anti-Angiogenic Factors
Source: PLoS One. 2015 May 7;10(5):e0126190. doi: 10.1371/journal.pone.0126190 (PMC4423857; doi:10.1371/journal.pone.0126190)
Supplement: S3 Table — Multiple regression analysis was used to assess the influence of independent predictors such as: age, BMI, triglycerides and LDL on serum levels of angiogenin. (DOC) [file pone.0126190.s006.doc]

**S3 Table. Assessment of the impact of age, BMI and serum lipid levels on serum angiogenin concentration**

Multiple regression analysis was used to assess the influence of independent predictors such as: age, BMI, triglycerides and LDL on serum levels of angiogenin.

| Variable | *β* | *p value* |
| --- | --- | --- |
| Age | 0,184 | 0,051 |
| BMI | 0,147 | 0,150 |
| TG | 0,171 | 0,096 |
| LDL | 0,077 | 0,415 |

BMI=body mass index, TG=triglycerides, LDL=low-density lipoprotein
