# Supplementary material for: Arterial Hypertension Is Characterized by Imbalance of Pro-Angiogenic versus Anti-Angiogenic Factors
Source: PLoS One. 2015 May 7;10(5):e0126190. doi: 10.1371/journal.pone.0126190 (PMC4423857; doi:10.1371/journal.pone.0126190)
Supplement: S5 Table — Multiple regression analysis was used to assess the influence of independent predictors such as: age, BMI, triglycerides and LDL on serum levels of endostatin. (DOC) [file pone.0126190.s008.doc]

**S5 Table. Assessment of the impact of age, BMI and serum lipid levels on serum endostatin concentration**

Multiple regression analysis was used to assess the influence of independent predictors such as: age, BMI, triglycerides and LDL on serum levels of endostatin.

| Variable | *β* | *P value* |
| --- | --- | --- |
| Age | 0,379 | 4x10^-4^* |
| BMI | -0,021 | 0,829 |
| TG | 0,319 | 0,001* |
| LDL | -0,150 | 0,094 |

BMI=body mass index, TG=triglycerides, LDL=low-density lipoprotein, statistical significance (p<0.05) is marked with “*”
